# Using Artificial Intelligence to Enhance Ongoing Psychological Interventions for Emotional Problems in Real- or Close to Real-Time: A Systematic Review

**DOI:** 10.3390/ijerph19137737

**Published:** 2022-06-24

**Authors:** Patricia Gual-Montolio, Irene Jaén, Verónica Martínez-Borba, Diana Castilla, Carlos Suso-Ribera

**Affiliations:** 1 Department of Basic and Clinical Psychology and Psychobiology, Jaume I University, 12071 Castellon de la Plana, Spain; gualp@uji.es (P.G.-M.); ijaen@uji.es (I.J.); susor@uji.es (C.S.-R.); 2 Instituto de Investigación Sanitaria de Aragón, 50009 Zaragoza, Spain; 3 Department of Personality, Assessment, and Psychological Treatments, Universidad de Valencia, 46010 Valencia, Spain; diana.castilla@uv.es; 4 CIBER of Physiopathology of Obesity and Nutrition (CIBERON), 28029 Madrid, Spain

**Keywords:** psychotherapy, artificial intelligence, emotional problems, systematic review

## Abstract

Emotional disorders are the most common mental disorders globally. Psychological treatments have been found to be useful for a significant number of cases, but up to 40% of patients do not respond to psychotherapy as expected. Artificial intelligence (AI) methods might enhance psychotherapy by providing therapists and patients with real- or close to real-time recommendations according to the patient’s response to treatment. The goal of this investigation is to systematically review the evidence on the use of AI-based methods to enhance outcomes in psychological interventions in real-time or close to real-time. The search included studies indexed in the electronic databases Scopus, Pubmed, Web of Science, and Cochrane Library. The terms used for the electronic search included variations of the words “psychotherapy”, “artificial intelligence”, and “emotional disorders”. From the 85 full texts assessed, only 10 studies met our eligibility criteria. In these, the most frequently used AI technique was conversational AI agents, which are chatbots based on software that can be accessed online with a computer or a smartphone. Overall, the reviewed investigations indicated significant positive consequences of using AI to enhance psychotherapy and reduce clinical symptomatology. Additionally, most studies reported high satisfaction, engagement, and retention rates when implementing AI to enhance psychotherapy in real- or close to real-time. Despite the potential of AI to make interventions more flexible and tailored to patients’ needs, more methodologically robust studies are needed.

## 1. Introduction

### 1.1. The Challenge of Treating Emotional Problems

Emotional problems occur very frequently in the population. In particular, depressive and anxiety disorders, also known as emotional disorders (EDs), are the most common psychological disorders and represent a global mental health problem because of their alarming prevalence rates and associated consequences in terms of economic costs and emotional suffering [1,2,3]. For example, lifetime prevalence of EDs in Europe has been argued to be as high as 44.5% in women and up to 26.5% in men [4] and researchers estimate that more than 38% of Europeans suffer from a mental disorder every year [5]. As a consequence, EDs have become one of the most common problems in primary care services globally [6,7]. Some studies suggest that these conditions are already present in up to 20% of primary care consultations [8,9,10] and research estimates that EDs will become the leading cause of disability worldwide in 2030, even ahead of musculoskeletal problems [11,12].

This alarmingly high prevalence of EDs has important consequences for our health systems and for the care received by persons with EDs. In many European countries, including high-income countries that have well-developed health care systems and universal health coverage, the availability of public, free-of-cost psychological treatments for mental health problems is insufficient or very difficult to access. In fact, studies globally indicate that often only one in four individuals with EDs receives psychological treatment and, in many cases, these interventions are not evidence-based [13,14]. Barriers for mental care include long waiting lists, co-payment, and inadequate resources, which pushes individuals with EDs who can afford it to the private system [15,16,17,18]. In addition, in some European countries public coverage includes psychiatric but not psychological treatment, even though guidelines indicate that several psychological treatments, especially Cognitive Behavioral Therapy (CBT), have the highest level of evidence and may be considered as initial interventions for people with mild-to-moderate depression or anxiety [19,20,21,22,23].

As if the above were not enough, the current coronavirus pandemic has further evidenced the importance of mental health, as well as the risk of its inefficient management for countries and individuals [24,25,26]. For example, some studies suggest that the prevalence of emotional problems may have tripled during the pandemic [27,28,29], so a coordinated response by governments and the global health community is more necessary than ever. We urgently need evidence-based and scalable prevention and treatment programs that can be broadly disseminated and universally accessible to cover the maximum possible of the population with EDs. It is crucial to change the current model of mental care to make it sustainable and reachable [30].

### 1.2. The Role of Information and Communication Technologies (ICTs) in the Treatment of People with EDs

The interest in finding more accessible and evidence-based forms of psychotherapy for EDs is not new [31]. For example, the British National Health Service has been developing and implementing the Improving Access to Psychological Therapies initiative for years, which uses a stepped model of care. Specifically, less severe patients (e.g., those with mild-to-moderate depression, panic disorder, generalized anxiety disorder, or obsessive-compulsive disorder) receive self-applied internet treatments before being treated with specialized and expensive modalities (i.e., individual or group face-to-face therapy), which are offered once the first ones fail or when the patients present a more severe condition [32,33].

Although stepped models of care have already achieved some milestones, the technological advances that have emerged in recent years (the explosion of the internet and smartphones) are largely responsible for the acceleration of some changes in mental care that otherwise would have been almost unthinkable. For example, more automated and easier ways of disseminating and administering psychotherapy have emerged over the past two decades. These treatments include, for example, psychological interventions that are provided in part or completely using phone calls, but of particular interest are self-administered treatments delivered through the internet (web-based), smartphone apps, or a combination of these [34,35].

Among the range of internet psychological treatments, most research has focused on the effectiveness of CBT delivered through a computer or mobile device (iCBT). iCBT has already demonstrated its efficacy in more than 100 randomized trials [36,37], even when compared with active face-to-face treatments, revealing that both options are generally just as effective [38,39,40,41]. Similar results have been found when these treatments have been aimed at people with chronic somatic diseases [42], so these self-applied treatments are promising alternatives to traditional face-to-face interventions also among people with chronic diseases. The inclusion of these technologies in psychological care has also helped to overcome some barriers and obstacles to psychological treatment, such as stigma, lack of anonymity, waiting lists, the high economic costs associated with face-to-face therapy, and the need to travel, among others [43].

Self-administered online treatments can reach the patient in a more economical and immediate way and are accessible to people who would otherwise experience difficulties when trying to receive treatment (e.g., due to long geographical distances to health centers, limited financial resources, or lack of time). Therefore, these interventions are feasible alternatives from an economic point of view, since they allow the dissemination of evidence-based treatments at low cost [44].

### 1.3. Limitations of (Internet-Based) Psychological Treatments for EDs and Contributions of Artificial Intelligence

Traditionally, research has assumed that certain treatments will be more effective than others for particular conditions, which is partly true [45,46,47]. However, clinical trials have also shown that psychotherapy works on average but not for a significant percentage of individuals [48]. Specifically, up to 40% of patients do not appear to improve or only partially respond to psychological interventions [49,50]. As Grimley Evans pointed out in the mid-1990s [51], while “administrators and researchers may be happy that treatments work on average, patients expect healthcare professionals to do better than that”. For the field of psychotherapy to advance, we should change the focus from the study of overall treatment effectiveness for a heterogeneous group of people, to evaluating the particular therapeutic presentations that fit best with each patient [52]. For example, an approach based on symptom reduction has been shown to be more effective for patients with externalizing problems, while an insight-based therapy is generally more effective for patients with internalizing problems [53], which would indicate that not all psychotherapies will be equally effective across individuals. The iconic question by Gordon Paul “What treatment, administered by whom, is most effective for this person with that specific problem and under what circumstances?” is still relevant today more than five decades later [54].

Unfortunately, technology-supported psychological interventions for the self-management of EDs have consisted of fixed protocols that are universally administered, thus ignoring patients’ specific needs and evolution during the intervention [55,56]. Even for evidence-based psychotherapies carried out face-to-face, it is not recommended to adhere strictly to more or less manualized treatment protocols nor to give predetermined responses to patient behaviors [57]. Therefore, some authors support a model of flexibility in fidelity as opposed to rigid, manualized treatments [58]. Ecological momentary interventions (EMIs) supported by artificial intelligence could facilitate this model change.

EMIs consist of making real-time or very short-term adjustments during the psychotherapeutic process based on the information received from the patient during ecological and momentary assessments, also known as EMAs [59,60]. These interventions can be used as a complement to existing psychological therapies provided by a therapist or they can be implemented as an independent intervention, for example in internet treatments [61], as well as to monitor and encourage participants to actively perform tasks (for example, by sending a notification on the mobile phone that encourages patients to do the tasks; [60]). This can be a very important mechanism to improve not only treatment effectiveness but also adherence in self-applied treatments using technology.

EMIs can play a key role in the future of psychological therapy by providing patients with timely therapeutic recommendations or instructions when problems arise, rather than later during face-to-face appointments. It has been argued that this reduces patient suffering, improves treatment effectiveness, and reduces treatment costs [62,63], which makes this a very suitable methodology to be implemented in the next generation of psychological treatments for EDs.

Although EMIs represent an important advance compared to episodic face-to-face interventions and even self-applied psychotherapy because of their traditional rigidity, it is known that not all people respond in the same way to psychological treatments [64], including the ones supported by technology [65]. That is why, for some years now, there has been a growing interest in understanding the reasons why an intervention works for some patients and not for others [66]. Artificial intelligence can assist in this endeavor and facilitate the decision-making process in real- or close to real-time by analyzing large amounts of complex data and providing the therapist with the relevant information for optimal management of an individual’s problem [67].

The term Artificial Intelligence (AI) was coined by John McCarthy when referring to the ability of a machine to imitate or simulate human-like functions, such as reasoning, learning, interaction, decision-making, adaptations, and sensory understanding through technology [68,69]. For example, a machine could cleverly manage the interaction with the user, as in conversational chatbots. Thus, chatbots can be used to complement the work of clinicians for those in need of mental health services when there are insufficient resources [70]. However, the definition for AI has changed in recent times and AI now refers to agents or calculators of historical records that may create prediction models, which helps to make very complex decisions. AI, for example, provides computer systems the ability to learn automatically through self-learning algorithms and improve from experience to maximize the precision of the processes [71]. AI has begun to be used in psychology, for example, to retrospectively evaluate which factors predict a better response to psychological treatment once it has finished [72,73]. It is less frequent, however, to use AI for the real- or close to real-time improvement of psychological interventions [74,75]. The use of AI can be especially useful in the context of a self-applied psychological therapy using technology since it could help investigate which EMIs worked best for which patients to personalize interventions. The aim of the present work is to review the evidence on the use of AI to enhance outcomes in psychotherapy in real-time or as close as possible to real-time.

The aim of the present work is to review the evidence about the use of AI to enhance outcomes in psychotherapy in real-time or as close as possible to real-time. In doing so, we reviewed (1) the characteristics of AI procedures used and (2) the evidence regarding the feasibility, acceptability, and clinical effectiveness (i.e., ability to lead to changes in symptomatology) of AI for psychotherapy.

## 2. Materials and Methods

This systematic review followed the guidelines of the Preferred Reporting Items for Systematic Reviews and Meta-Analyses (PRISMA) [76]. This study was retrospectively registered on PROSPERO (CRD42022245856) with some adaptations to a preliminary version submitted to the platform that resulted from insights and novel knowledge acquired during the review process. The PROSPERO record cannot be updated at this stage, but the deviations will be detailed during the following lines in the corresponding sections.

### 2.1. Identification and Selection of Studies

Electronic searches were conducted using Scopus, Pubmed, Web of Science, and Cochrane Library. The last search was conducted on 30 March 2022.

Following the PICOS framework (P = participants; I = interventions; C = comparison; O = outcomes; and S = study design), two investigators independently analyzed the titles and abstracts of the retrieved studies to exclude those unrelated to the review topic. Full texts were retrieved for a final evaluation for potentially relevant articles. The selection process was consensus-based. When a consensus was not reached, a third reviewer was included. The references from the articles included were also screened by two independent investigators to identify potentially important studies that were not retrieved in the electronic search.

Inclusion criteria:

- An AI method is implemented.
- The target population is people with emotional problems. We included volunteers with daily stressors or emotional problems without a formal assessment of emotional disorder since we were interested in assessing changes in emotional symptomatology, regardless of the severity of the emotional problem. This was updated from the original PROSPERO registration since a number of studies using AI to enhance psychotherapy included this population that was originally ignored from our initial review plan.
- Psychological treatment is the main intervention.
- AI is implemented to improve an ongoing intervention.

Exclusion criteria:

- Data obtained with AI are not used to make changes in the treatment during therapy (e.g., predictors of treatment efficacy are evaluated at the end of an intervention for a group of individuals). This was not specifically stated in the original PROSPERO registration because of unintentional omission, but it represents an important exclusion criterion that was implicitly taken into account by the reviewers when selecting the included studies.
- The study is a protocol with no results available.

### 2.2. Search and Screening

The search strategy included variations of the terms “psychotherapy”, “artificial intelligence”, and “emotional disorders” (See Supplementary File S1 for the complete list of search terms and combinations). Due to the diversity of terms, a broad search strategy of terms was used. Synonyms, abbreviations, and spelling variations were identified for the three concepts and combined in the search using the “OR” Boolean operator, with non-synonymous concepts combined using “AND”. These terms were searched in titles and abstracts. The references of included studies and relevant systematic reviews were searched to identify studies that were missed during the literature search. The terms were agreed by all the study authors and the search was then conducted by CSR. Half of the articles were screened by CSR and PGM, while the other half were screened by IJ and VMB.

### 2.3. Data Extraction

A pre-designed data extraction sheet was used. This sheet contained general study information (e.g., authors and year of publication), sample characteristics (e.g., age and sex/gender), design/methodological characteristics, including risk of bias assessment, information about the treatment, including characteristics of the AI method, and primary and secondary outcomes.

### 2.4. Risk of Bias Assessment

The quality and risk of bias of the eligible studies was assessed using the study quality assessment tools from the National Heart Lung and Blood Institute (NHLBI, https://www.nhlbi.nih.gov/health-topics/study-quality-assessment-tools, accessed on 10 April 2022), which includes six types of studies and specific criteria according to the study design (i.e., controlled intervention studies, systematic reviews and meta-analyses, observational cohort and cross-sectional studies; case-control studies, before–after studies with no control group, and case series studies). In the original PROSPERO registration, we anticipated the use of the ROBINS-I (https://methods.cochrane.org/methods-cochrane/robins-i-tool, accessed on 5 April 2022) quality assessment tool. However, after revising other systematic reviews, the authors decided to change to the NHLBI because it provides specific quality criteria for a wide range of study designs, which is important for the current review considering the different designs included. The total quality scores are ranged from 9 to 14 points depending on the study design. It allows researchers to give an overall rating of “good”, “fair”, or “poor” for each study. All the studies were independently rated for quality by three reviewers (IJ, PGM, and VMB), who reviewed the quality of papers in pairs. The Kappa coefficient was 0.783 (SE = 0.201; 95% CI, 0.388, 1.000), thus suggesting a substantial agreement. Disagreements were resolved through discussion to reach consensus with a fourth reviewer (CSR).

## 3. Results

### 3.1. Selection and Inclusion of Studies

**Figure 1:**
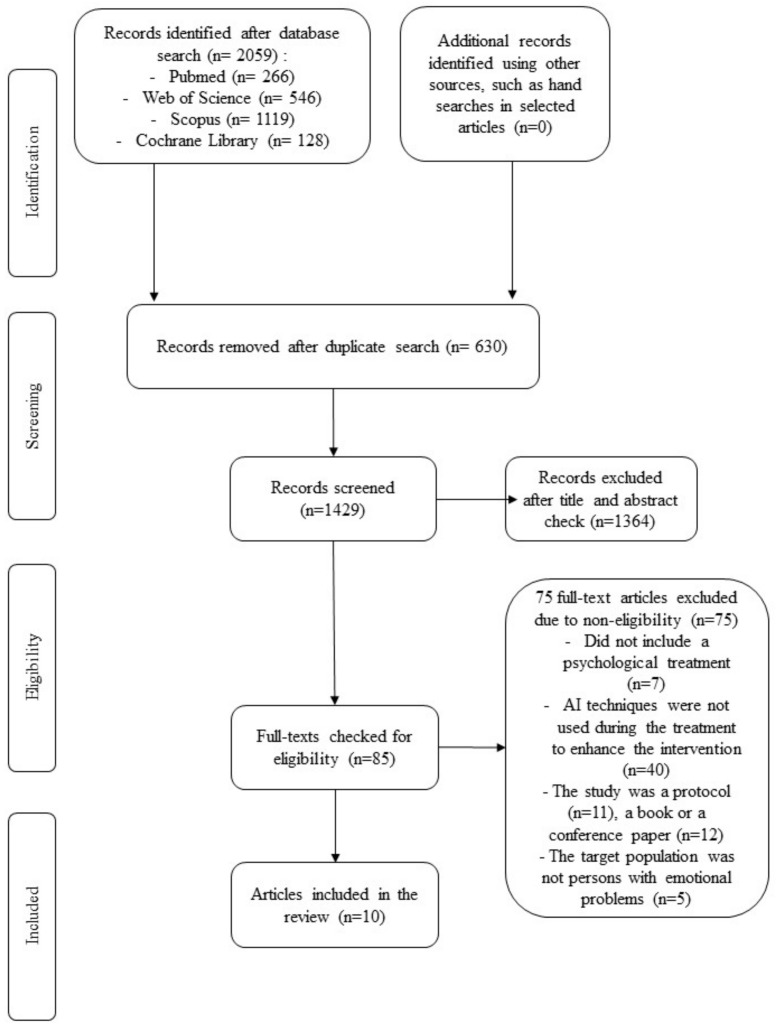
Selection progress of the review.

The search in the four databases generated a total of 2059 studies (PubMed = 266; Web of Science = 546; Scopus = 1119; and Cochrane Library = 128). After eliminating the duplicates (*n* = 630), a total of 1429 records were screened by four independent researchers in groups of two (PGM and CSR screened half of the records and IJ and VMB reviewed the other half) based on the titles and abstracts. After the exclusions, 85 full-text versions were assessed for eligibility and were excluded if they did not include a psychological treatment (*n* = 7), AI techniques were not used during the treatment to enhance the intervention (*n* = 40), the study was a protocol (*n* = 11), the study was a book or a conference paper (*n* = 12), or the target population was not persons with emotional problems (*n* = 5). The study selection process in presented in the PRISMA flowchart (Figure 1).

In the eligibility assessment, inter-rater agreement was calculated using Cohen’s Kappa. The inter-rater agreement was excellent both when screening records from the first time (Kappa = 0.936, SE = 0.023; 95% CI = [0.891, 0.980]) and when selecting included studies (Kappa = 0.887, SE = 0.079; 95% CI = [0.733, 1.000]).

### 3.2. Characteristics of Included Studies

#### 3.2.1. Country Where the Study Was Conducted

The characteristics of included studies are shown in Table 1. Of the 10 studies included in the systematic review, 3 were published in the USA [77,78,79], with the remaining studies being published in the United Kingdom (*n* = 2; [80,81]), Germany (*n* = 1; [82]), Switzerland (*n* = 1; [83]), Korea (*n* = 1; [84]), Kenya (*n* = 1; [85]), and China (*n* = 1; [86]).

#### 3.2.2. Target Populations and Sample Sizes

Regarding the type of populations included, most studies focused on people with depressive symptoms (*n* = 7, [77,81,82,83,84,85,86]). Some investigations also included population with affective symptoms together with PTSD, anxiety problems, and other diagnoses (*n* = 1; [82]), manic/hypomanic episodes (*n* = 1; [84]), or stress (*n* = 1, [79]). Two studies targeted volunteers with daily stressors that would impact their emotional status (*n* = 2; [78,80]). The sample sizes of the included investigations ranged from 8 to 1234 participants.

#### 3.2.3. Design and Treatment

In terms of design, four studies were pre–post investigations [77,81,82,83], four were randomized controlled trials (RCTs; [78,79,80,86]), one study was a case-control study ([84]), and one was a non-concurrent multiple baseline single group [85].

Regarding the treatments offered in the included studies, most studies provided only cognitive behavioral therapy (CBT; *n* = 4; [82,83,85,86]). Two investigations offered behavioral guidance interventions (*n* = 2; [77,84]), one study offered a problem-solving intervention based on method of levels [80], and another provided stress management micro-interventions, in the form of positive psychology, cognitive behavioral, meta-cognitive, and somatic treatments [79]. Other investigations included CBT together with other interventions, that is dialectical-behavioral therapy, motivational interviewing, positive psychology, behavioral reinforcement, mindfulness-based therapy, acceptance and commitment therapy, interpersonal psychotherapy, metacognitive treatment, somatic intervention, emotionally focused therapy, and self-compassion therapy [78,81].

#### 3.2.4. Primary Outcomes

The primary outcomes also differed across investigations. Most studies included the measures of depression and anxiety, namely the Patient Health Questionnaire-9 (*n* = 7; [77,78,79,81,83,85,86]) and the Generalized Anxiety Disorder-7 (*n* = 3; [77,78,86]) as primary measures in their investigations. Other primary outcomes included the Coping Strategies Questionnaire [79], the Outcome Questionnaire [82], the Assessment for Signal Clients [82], the Affective Style Questionnaire [82], the Hopkins Symptom Checklist [82], the Quick Inventory of Depression Symptom-clinician rated [77], the daily mood ratings [84], the problem-related distress ratings [80], and positive and negative affect schedule [78,86].

#### 3.2.5. Characteristics of Artificial Intelligence procedures

Consistent with the two broad dimensions of AI presented in the introduction (i.e., chatbots or calculators), the included studies were classified in this manuscript accordingly [68,69].

Five of the reviewed studies used conversational AI agents as AI method to provide therapeutic guidance in real-time or close to real-time. These chatbots were based on a software that could be accessed online with a computer [80] or a smartphone [78,81,86]. Studies presented differences in the time of chatbot availability, which ranged from 15 min to deal with specific daily problems [80] to CBT-based conversational interventions available anytime [78,81,85,86]. All the chatbots used written language to interact with the system and the participant, while one investigation also included the recognition of voice messages [86].

For example, in one study [80], the authors developed the chatbot Manage Your Life Online (MYLO), a computer-based intervention that used AI to create questions and answers (conversations) for problem-solving with the participants. In this study, AI was used to analyze the participant’s input and well-being status (e.g., anger) and then facilitate more awareness and problem-solving accordingly, while comparing them to another group using ELIZA, a less complex AI intervention. In another investigation, the authors implemented a smartphone app, called Tess [78], that used AI to examine natural conversations of the participants, as in the previous study. Here, however, AI was used to identify and interpret the participants’ emotions in the text messages and then deliver brief personalized interventions or reminders. In this study, there was a comparison between participants that use Tess for 2 weeks, 4 weeks, or a control group that use an electronic eBook for depression. Additionally, the Tess project had a panel where the professionals could view the participants’ interactions with Tess. A similar example was the app Zuri (Tess in Kenya), which was used to deliver text messages to the participants to assess their emotional states and offer brief psychological modules depending on their conversations with the AI chatbot [85].

Another study used Wysa, a conversational AI agent to promote positive self-expression and well-being [81]. Here, AI was used to analyze emotions that users expressed through conversations and to deliver psychological skills based on CBT or Dialectival Behavioral Therapy. Finally, in another investigation, a chatbot called XiaoNan was used for conversational intentions to alleviate depression [86]. Here, both text and voice messages were used. Then, three machine learning models processed the information from the user, labeled the input, and generated responses based on CBT principles. In this study, comparisons were made between the use of XiaoNan and a control group who receive bibliotherapy.

The remaining studies (*n* = 5) implemented AI to predict patient mood based on behavioral and self-reported data and provided feedback to the participants to promote behavioral change (i.e., second type of AI). For example, a study [84] used a circadian rhythm-based algorithm based on data obtained with a wearable activity tracker to predict changes in health and mood over the following 3 days and provided feedback messages with these predictions and warning alerts (e.g., “Your life rhythm is irregular”). This was done to encourage the participant to engage in positive behavioral changes. Additionally, another study [77] used decision trees that predicted personal states based on information from mobile phone sensors and the participant’s reported data using ecological momentary assessment. These predictions were shared with the participants to reinforce positive changes or to suggest using a behavioral activation tool. Like in the previous case, two studies also used AI to make predictions based on self-reported and/or physical data, in this case to personalize upcoming micro-interventions [79,83]. In particular, both studies used information from a smartphone to predict which micro intervention most effectively reduced stress [79] or depressive symptoms [83] to proposed changes close in time or in real-time. A similar strategy, in this case comparing problem-solving, motivation-oriented strategies, or a combination of both, was followed by another study [82]. In addition, in this latter study the authors also used AI to obtain a prediction of the individuals’ drop-out risk and therefore recommend therapist attention in real-time or close to real-time.

#### 3.2.6. Changes in Clinical Symptomatology

Most studies focused their outcomes on depressive (*n* = 10; [77,78,79,80,81,82,83,84,85,86]) or anxiety symptoms (*n* = 5; [77,78,80,82,86]). Additional mental health conditions and symptoms included manic/hypomanic episodes (*n* = 1; [84]), psychological functioning (subjective discomfort, interpersonal relationships and social performance; *n* = 1; [82]), stress reduction (*n* = 1; [79]), and affect improvement (*n* = 2; [78,86]). Some investigations also focused on improving psychological skills, such as constructive coping (*n* = 1; [79]), problem solving (*n* = 1; [80]), or behavioral changes (*n* = 1; [84]).

As reported in Table 2, almost all studies found positive effects when implementing AI to reduce psychopathology. Both pre-post (*n* = 4; [77,81,82,83]) and controlled studies (*n* = 5; [78,79,80,84,86]) found a reduction in depressive symptoms after using a tool with integrated AI. One of the pre-post investigations also explored differences in outcomes attending to engagement levels [81]. The authors reported that high users (i.e., participants who responded to the two pre-post assessments and one additional in-between evaluation) showed a significantly greater improvement compared with low users, that is, participants engaging only in the two pre-post assessments [81].

In addition to changes in depression, both controlled (*n* = 3; [78,80,86]) and uncontrolled (*n* = 2; [77,82]) studies including a measure of anxiety evidenced a reduction in this symptom when using AI. Some studies with controlled interventions compared an AI chatbot that delivered psychological interventions with either an active control group without AI [78,86] or with a less complex AI chatbot [80]. In another controlled study [79], comparisons were made between groups in which AI was used to recommend individual interventions (e.g., personalized interventions) or interventions were randomly chosen without using AI. In another study, a comparison was made between a group using a circadian rhythm for mood app while receiving feedback from their status and without receiving their feedback [84]. The findings in relation to the remaining outcomes, namely the severity and duration of manic/hypomanic episodes severity [84], overall psychological functioning [82], stress levels [79,80], affect [78], behavioral changes [84], constructive coping [79], and problem resolution [80], were also positive when implementing AI. Only one study failed to reveal significant changes in affect after implementing an AI-based chatbot intervention [86].

#### 3.2.7. Engagement

Different studies have analyzed how users adhere to the different AI-based programs (Table 2). In general terms, sample retention ranged from approximately 20% [79,86] to almost 52% [85]. For example, the proportion of participants who completed at least one of the wellness tools proposed by one study [81] was around 60%, while the participants in other study [77] completed 53.3% of sessions.

Regarding continuous assessments retention, a study [81] found high longitudinal retention rates in the short term (most participants were engaged during more than 4 days), whereas three studies [77,79,86] evidenced a reduction in engagement over the course of the treatment. In one study [77], mobile phone training dramatically decreased from the first week of the intervention (mean = 15.3 training episodes; SD = 8.3) to the eighth week (mean = 4.8 training episodes; SD = 4.6). Only two studies compared drop-out rates as a function of the condition’s assignment (AI vs Random technique recommendations/control group). The results indicated that both groups showed comparable drop-out rates [79,86]

The factors that may have contributed to low engagement according to the included studies were: having children, problems at work, or illness [79], certain personality traits (severe histrionic traits and not obsessive), difficulties on interpersonal relationships, poor treatment expectations, lack of university entrance qualifications [82], and being pregnant or employed [85]. Inconsistent results were found for marital status and clinical symptomatology. One study [79] found that married participants were at risk for drop-out, while another study [85] indicated that married users were more engaged with the program. Similarly, results in one study [82] revealed that less severe anxiety and depressive symptoms were related with higher drop-out, and another study indicated that more severe depressive symptoms were in fact a risk factor for poor engagement [85].

#### 3.2.8. Satisfaction with AI

As reported in Table 2, the participants generally rated AI interventions to be helpful [81] and satisfactory [77]. Users of AI systems reported greater satisfaction, learning skills, and therapeutic alliance than users with non-AI-based programs, such as eBooks, bibliotherapy, or text-based programs [78,80,86]. AI users highlighted positive aspects of including AI, such as accessibility and empathy [78,86]. AI users also stated that chatbots were friendly, interesting, educational, and interactive [86]. In terms of confidence, users had a positive attitude toward the use of AI and trusted this system [85].

Some studies also indicated problems associated with the use of AI-based programs. Two main categories emerged in the studies. One was related to technological issues, such as loss of connectivity, battery life, and phone freezing [77]. The other referred to human vs programmed interactions and included problems such as the limitation of non-natural conversations, some degree of impersonality, the rigidity of some response patterns, repetitiveness, irrelevance of some interactions, perceived lack of specificity of contents, poor interactivity, high simplicity of interactions, or existence of misunderstandings, that is, AI chatbots not understanding users’ responses or giving unexpected responses [78,86].

#### 3.2.9. Risk of Bias Assessment

As observed in Table 3, Table 4, Table 5 and Table 6, studies included in this review could be placed in four of the categories proposed by the National Heart Lung and Blood Institute. As observed in Table 3, Table 4, Table 5 and Table 6, studies included in this review could be placed in four of the categories proposed by the National Heart Lung and Blood Institute, namely, before-after studies, case series studies, case-control studies, and controlled intervention studies.

Overall, the four studies classified as before-after studies had a “good” quality, with total scores ranging from 8 to 10 points out of a maximum of 12 points [77,81,82,83]. Qualitative flaws were mostly found in missing power or effect size [77,83] and no blinding of assessors [77,82,83]. Additionally, two of the studies suffered from potential bias in their loss to follow-up [82,83]. The study classified as a case series study (pre-pilot single-case experimental design) could also be rated as a “good” quality investigation because it obtained 8 points of a maximum of 9 [85].

In the investigation classified as a case-control study [84], the main issues were related the lack of sample size justification, no blinding of assessors, and an unclear participant selection. Thus, it met only seven criteria of a maximum of 12, so its quality could only be rated as “fair”.

Finally, one of the four studies classified as a controlled intervention [79] was rated as having a “poor” quality (2 out of 14 points) as it did not follow most of the criteria for controlled interventions (i.e., randomization, large sample size, blind allocation, or assessment). One of the remaining three studies [80] was rated as “fair” with an 8 out of 14, as the condition to which each participant was allocated was not masked for the providers, drop-out rates at endpoints were relatively high, and the authors did not use valid assessment procedures. The remaining controlled intervention studies were classified as “good” quality investigations as they met most of the criteria [78,86].

## 4. Discussion

This study aimed to systematically review the existing literature regarding the use of AI to improve ongoing psychological interventions for emotional problems in real-time or close to real-time. The importance of the topic lies in the fact that AI might allow us to rapidly improve and personalize ongoing psychological interventions during the therapeutic process, as well as to enhance patient response to them. Traditionally, the combination of AI and psychotherapy has been used to retrospectively evaluate large amounts of data once a psychological intervention has finished (i.e., to evaluate the outcome data that can predict a better response to a given psychological treatment or to identify risks for becoming a not-on-track patient once the treatment ended) [87]. However, AI may allow us to investigate which psychological interventions and EMIs work best for which patients to tailor psychotherapy to patients’ needs in real- or close to real-time during the therapeutic process [71].

In this systematic review, 85 full-text studies assessed for eligibility after reviewing more than 1400 titles and abstracts and only 10 studies met our inclusion criteria, which suggests that this is an infrequently researched topic. Moreover, from the included studies, the sample sizes, study design, psychological interventions delivered, and type of AI technique used clearly varied across studies, which again suggests that this is a field that requires more research, replicability, and generalizability to obtain robust findings. In particular, sufficiently powered RCTs with a-priori sample size calculations would be preferable because these designs are considered to be superior to uncontrolled studies because of their higher internal validity and robustness to explain causal relationships. Note that, in the included studies, AI was applied to a relatively heterogenous number of participants, which ranged from 8 to 1234 participants, and most investigations did not report sample size calculation processes, which negatively impacts the reliability, replicability, and generalizability of findings.

Regarding the application of AI for psychological interventions, our systematic review found that several AI methods have been applied so far. Our results also evidenced that the AI classifiers and algorithms used clearly varied across studies. While all AI applications used might lie within the definition proposed by Russell and Norvig [88] of agents that receive environmental percepts and respond to affect such an environment, the two approaches used so far clearly differ from one another. The most frequently used AI technique was conversational AI agents (a text-based program using AI), which are chatbots based on software that can be accessed online with a computer or a smartphone. In some cases, however, AI was also used to predict patient responses and changes based on patients’ data and to provide feedback messages to them with the alerts created to promote a behavioral change (i.e., measurement-based care). A reduced number of studies implemented other techniques/algorithms, such as reinforcement learning algorithms, decision trees, random forest algorithm, and support vector machines, again supporting the idea that this field requires further development.

An important finding regarding the use of AI for the improvement of ongoing psychotherapy was that, in general, all the investigations indicated that AI has some potential to enhance psychotherapy and help reduce clinical symptomatology (e.g., depressive symptoms, anxiety symptoms, psychological functioning, and stress, among others) or prevent mood episodes (e.g., manic/hypomanic episodes). While these findings should be interpreted with caution due to the scarce number of existing investigations, particularly high-quality ones (e.g., controlled studies), the heterogeneity of included populations, and the qualitative flaws found in the blinding procedure, sample size justification, and the overall drop-out of the included studies, the results are promising regarding the use of AI to personalize and tailor psychological interventions. Congruently, a recent study proposed that psychotherapy can be supported by computation and claimed that AI should be understood as an additional resource for therapeutic work apart from the existing ones [69].

An additional interesting finding was that most studies reported high satisfaction and retention rates when implementing AI methodology to enhance psychotherapy in real-time and indicated that engagement with the AI system was associated with greater improvement in symptomatology. In general, patients found AI-based psychological programs to be helpful and generally indicated that the AI system met their needs. Patients mentioned that some of their preferred characteristics of AI programs, particularly chatbots, included their accessibility, empathy, confidence, and friendliness. A few investigations, however, indicated a decrease in engagement during the treatment and revealed some factors that might negatively impact engagement (e.g., having children, problems at work, personality traits, and low treatment expectations). Similarly, some investigations reported some technical (e.g., battery life, burdensome, and loss of connectivity) and emotional (e.g., loss of human interactions, loss of natural conversational, or impersonality) challenges when receiving AI-supported psychotherapy. These barriers point to further developments that need to be considered in future applications using AI. Future efforts in this direction should be made to guarantee that AI development considers patients’ opinions and also that AI-based technologies are developed in a safe, trustworthy and overseen context [89].

In recent years, extensions and innovations of psychotherapy, such as treatments enhanced by ICT or AI, have been developed to facilitate the dissemination of psychotherapy considering the great number of limitations in existing resources of mental health care (i.e., long waiting lists in public health systems and inadequate resources) [15,16,17]. Even though AI is a great option to enhance effectiveness and make psychotherapy more accessible, it is crucial to mention that this emerging field does not aim to replace the role of the psychotherapist [70,89,90]. The intention of incorporating AI in the field of psychotherapy is to reach as many people in need as possible (i.e., increase reach [44,70]), and to provide information on symptom progress over the course of psychological interventions to the therapists so that this information can be used to rapidly detect and react to problems that might occur during interventions (e.g., recovery trajectories that do not occur as expected during psychotherapy). AI is therefore an add-on tool for the therapeutic process [69]. The utility of AI algorithms lies in providing opportunities for tailoring treatments according to patients’ needs (physiological states, EMAs, and EMIs) and making current interventions more flexible. AI methods allow us to provide the best treatment for a particular patient with its idiosyncratic/idiographic information at the right time. Therefore, the use of AI as an add-on tool for ongoing psychological interventions may offer some potential for improving psychotherapy. Particularly, technological interventions in real-time or close to real-time, such as EMIs supported by AI to may make short-term adjustments during the psychotherapy process, may be an excellent alternative to current rigid psychological interventions. While the presented results are, overall, encouraging, especially to tailor and personalize treatments, more research is required in this field. We expect that the present work will offer researchers and clinicians with a concise summary of the current advances of the field and will inspire future research.

### Limitations

Limitations of this systematic review should be considered when interpreting the results. First, the heterogeneity on sample sizes, the measures used, and the methodologies implemented clearly impact the generalizability and robustness of the findings. Regarding the use of AI-based programs, most studies only tested the AI tools within the same sample, limiting the external validation and generalizability of the results. In addition, due to the heterogeneity of studies, in this review we could not discuss the types of algorithms or AI techniques that yielded the best performances. Finally, it is important to note that this manuscript is limited to the interpretations of the authors who conducted the review.

Another limitation is that no clear distinction was made in the included studies regarding the type of AI used (human-like AI and calculator AI) and the effect that this could have on its effectiveness because insufficient and too heterogeneous data were available to conduct a meta-analytic calculation of pooled effects.

While acknowledging these limitations, the findings of this review generally support the idea that the information provided by AI tools over the course of psychological interventions might help therapists clarify treatment processes, but also detect and rapidly react to changes in the patient trajectories when delivering psychotherapy. The reviewed studies verify that AI might make interventions more flexible and tailored to patients’ needs. Despite the potential of AI in psychotherapy, this review evidenced that the integration of AI to enhance psychological treatments in real-time is still very rare and methodologically robust studies are needed. For example, it will be important to determine the optimal use of this AI-based programs to detect changes in the patients’ symptomatology rapidly and efficiently and to adapt treatments to their needs. Additionally, there are some challenges regarding the use of the data from the AI tools used during psychotherapy as it is paramount to protect information from individuals [72]. With the implementation of AI in psychotherapy, however, this task becomes arduous, so emerging AI methods must be aware of this issue. This systematic review aimed to bridge some of the previous gaps to move the science of AI closer to the clinical practice of psychotherapy.

## 5. Conclusions

To conclude, this systematic review found preliminary support for the use of AI tools to enhance psychotherapy for emotional problems in real-time or close to real-time during the therapeutic process. The majority of identified studies have demonstrated the potential of using AI during an ongoing psychological intervention, especially by providing patients with rapid and personalized automated feedback and therapeutic guidance. However, while research to date shows some potential in this regard, further investigations are required to support the idea that AI may positively impact the job of psychotherapists by providing real-time or close to real-time information of patient progress and treatment recommendations.

## Figures and Tables

**Table 1:** Characteristics of the included studies.

References	Country	Sample Size	Study Design	Emotional Problem Evaluated	Psychological Intervention	Main Outcome	Type of AI Used
[77]	USA	8 Tx	Pre-post study (no control)	MDD	Multimodal intervention (behavioral activation approach)	MINI, QIDS-C, PHQ-9, GAD-7	The nearest neighbor Suppport Vector Machines and Random Forest Classifier
[78]	USA	74 (24 cont. + 2-week 24 Tx + 4-week 26 Tx)	Parallel group RCT (1:1)	College students with daily stressors	CBT and other interventions	PHQ-9, GAD-7, and PANAS	AI chatbot: conversational Tess app
[79]	USA	95 Tx (22 no selection-random intervention; 21 no selection-AI intervention; 26 selection-random; 26 selection-AI)	Controlled trial	Stress	Stress management micro-interventions (positive psychology, cognitive behavioral, meta-cognitive, and somatic) via app	PHQ-9 and CSQ	Reinforcement Learning algorithm
[80]	UK	171 (85 Tx MYLO program + 86 cont. ELIZA program)	RCT	Volunteers with daily stressors	Web-based problem-solving intervention	Problem-related distress	AI chatbot; Manage Your Life Online (MYLO)
[81]	UK	129 Tx	Quasi-experimental pre-post study	Self-report symptoms of MDD (nonclinical global population)	CBT together with other interventions via Wysa app	PHQ-9	AI-chatbot: conversational Wysa app
[82]	Germany	1234 Tx	Pre-post study (no control)	Affective and anxiety disorders (70%), and other disorders (30%)	CBT via Trier Treatment Navigator	OQ-30, ASC, ASQ, HSCL-11	Random Forest Algorithm.
[83]	Switzerland	126 Tx	Pre-post pilot study	Depressive symptoms	CBT intervention + MOSS app	PHQ-9	LASSO (least absolute shrinkage and selection operator)
[84]	Korea	43 Tx (10 CRM group + 33 non-CRM)	Prospective Case-control study	MDD, BD I and BD II	Feedback intervention of Behavioral guidance	Daily mood state on eMoodChart, mood episodes, circadian rhythm	Circadian rhythm-based algorithm based on data obtained with a wearable activity tracker
[85]	Kenya	41 Tx (pregnant women and new mothers)	Nonconcurrent Multiple baseline SG	Non-clinical Perinatal Depression	CBT (Healthy Moms adaptation)	PHQ-9, feelings, and mood	AI chatbot: conversational Zuri app, Kenyan version of Tess
[86]	China	83 university students (42 cont. + 41 Tx)	RCT (unblinded)	Depression	CBT intervention	PHQ-9, GAD-7, and PANAS	AI chatbot: conversational XioNan app)

Note: Tx, treatment group; AI, artificial intelligence; CRM, Circadian Rhythm for Mood; MYLO, method-of-level therapy program; ELIZA, client-centered therapy program; RCT, randomized controlled trial; SG, single group design; MDD, Major Depressive Disorder; BD, Bipolar Disorder; CBT, Cognitive-Behavioral Therapy; App, mobile application; MOL, method of levels; AI, Artificial Intelligence; PHQ-9, Patient Health Questionnaire-9; GAD-7, Generalized Anxiety Disorder-7; CSQ, Coping Strategies Questionnaire; OQ-30, Outcome Questionnaire; ASC, Assessment for Signal Clients; ASQ, Affective Style Questionnaire; HSCL-11, Hopkins Symptom Checklist; MINI, Mini-International Neuropsychiatric Interview; QIDS-C, Quick Inventory of Depression Symptom-clinician rated; PANAS, Positive Affect and Negative Affect Scale.

**Table 2:** Results of clinical symptoms, engagement, and satisfaction.

References	Results
[77]	**Clinical symptoms**: - Improvements were found in PHQ-9 (t = 7.02, B = −0.82, *p* < 0.001), GAD (t = 4.59, B = −0.71, *p* < 0.001) and QIDS-C scores (t = 8.22, B = −0.81, *p* < 0.001). - Participants became less likely to meet diagnostic criteria for Major Depressive Disorder (*Z* = 2.15, B = −0.65, *p* = 0.030). **Engagement**: - Seven participants completed all eight weeks of participation, one participant dropped out because of technical problems. - Participants completed 4.8/9 sessions on the website. - Participants engagement with the mobile phone gradually decreased across the intervention. **Satisfaction**: - Average satisfaction with the mobile phone was 5.71/7. - The most common problems were loss of connectivity, shortness of battery life, and phone freezing during use.
[78]	**Clinical symptoms**: - Control group reported higher PHQ-9 scores than group 1 (*p* = 0.020). - Control group reported higher GAD-7 scores than group 1 (*p* = 0.045) and group 2 (*p* = 0.020). - Statistically significant differences were found between control group and group 1 in PANAS scores (*p* = 0.030). **Satisfaction**: - Tess users reported higher satisfaction, and learning. - Best aspects of the bot were accessibility and empathy. - Worst aspects were limitations in natural conversations, such as not being able to understand responses, unexpected answers, and low interactivity.
[79]	**Clinical symptoms**: - Users in the AI group reported greater stress reduction [*t*(46) = 2.06, *p* = 0.020] and less depression than random condition (*p* = 0.06). - AI participants reported more constructive coping behaviors over time [F(1,16) = 4.4, *p* = 0.003]. **Engagement**: - No differences between groups were found in drop-out. - Drop-out = married, had children, had trouble at work or illness. - Sample retention = 21% (*n =* 20).
[80]	**Clinical symptoms**: - All participants reported further resolution of their problems from post-intervention to follow up [F(1,60) = 48.78, *p* < 0.001, *n*^2^ *=* 0.45). The overall program effect was not significant (F(1,60) = 2.49, n.s). However, the users of the MYLO program reported higher problem resolution [*t*(131.27) = 4.76, *p* < 0.001] at the post-intervention. ELIZA program users showed further problem resolution between the post-intervention and follow-up than MYLO program users. - Both groups reported improvement in distress [F(2,338) = 51.10, *p* < 0.001, *n*^2^ = 0.23]. There were no differences between the two groups (F(1,169) = 0.69, *p* = 0.41). - A reduction in the DASS was found over the three time-points [F(2,314) = 49.39, *p* < 0.001, *n*^2^ = 0.24]. There was no main effect of group [F(1,157) = 16, n.s]. **Satisfaction**: - MYLO users rated the program as more helpful than ELIZA users (F(1,60) = 12.98, *p* = 0.001, *η*^2^ *=* 0.18).
[81]	**Clinical symptoms**: - Within group differences: both comparison groups showed a significant reduction in PHQ-9 score (high users: W = 478.5, *p* < 0.001; low users: w = 32.5, *p* = 0.010). - Between-groups differences: high users showed higher improvement compared with low users (*p* = 0.03, effect size = 0.63, Cohen’s d = 0.47). **Engagement**: - 83.3% of high users actively used the app for more than 4 days on. - 59.7% (77/129) of users completed at least one wellness tool provided by the app (72 high users and 5 low users). **Satisfaction**: - 67.7% rated favourable experience (helpful and encourage); 32% rated less favourable experience (unhelpful and concerns).
[82]	**Clinical symptoms**: - From pre-treatment to session 10, patients treated with the recommended strategy (optimal) had a significantly higher effect size than patients treated with a non-recommended strategy (non-optimal) on the HSCL-11 (t = 1.01 = −1.99, *p* = 0.048); on the OQ-30 (t_201.01_ = −2.19, *p* = 0.029). **Engagement**: - Drop-out was predicted by higher impairment on the FEP-2, lower impairment on the HSCL-11, a more histrionic personality, higher impairment of interpersonal relationships, a less obsessive personality style, a lower therapist treatment expectation, and a lack of university entrance qualification (all *p* < 0.050).
[83]	**Clinical symptoms**: - Symptom severity change: significant differences in the PHQ-9 from t_0_ to t_6_ (*p* = 0.04) and from t_0_ to t_8_ (*p* = 0.01). - Comparison of vectors machines and random forest classifier: - The random forest classification showed the highest accuracy and specificity. - The support vector machine obtained higher sensitivity compared with the random forest classification.
[84]	**Clinical symptoms**: - CRM group presented fewer (β = 0.033, *p* = 0.03) and shorter (β = 0.005, *p* < 0.001) depressive episodes than non-CRM group. - CRM group had shorter manic/hypomanic episodes (β = 0.039, *p* < 0.0001), fewer (β = 0.026, *p* = 0.008) and shorter (β = 0.011, *p* < 0.001) total mood episodes than the non-CRM group. - Positive behavioral changes in CR amplitude, light exposure during daytime, and steps during daytime were found when alert feedback was provided (assuming 95% CIs, *p* < 0.05). - No significant differences between groups were found in sleep.
[85]	**Clinical symptoms**: - Mood improved by 7% over the average mood reported at baseline period (d = 0.17). **Engagement**: - Retention rate of 51.9%. - Less engagement: pregnant, greater depression symptoms, and employed outside. - More engagement: married and more educated women. **Satisfaction**: - Women had a positive attitude and expressed that they could trust the AI Zuri program.
[86]	**Clinical symptoms**: - Participants who received chatbot intervention showed an increased reduction of depressive (F = 22.89; *p* < 0.01; *d* = 0.83) and anxiety symptoms (F = 5.37; *p* = 0.02; *d* = 0.30) compared to bibliotherapy group. No significant differences were found in the reduction of positive and negative affect. **Engagement**: - Attrition rate of 24.1% (20/83). - There were no statistically significant differences between completers and participants who dropped out in sociodemographic or psychological factors. - The chatbot group showed a deceased adherence during the five assessment points. The bibliotherapy control group slightly increased adherence rates during the first 8 weeks (two assessment points). Differences between both comparison groups were not significant. **Satisfaction**: - Chatbot users showed higher therapeutic alliance than bibliotherapy control group (*t* = 7.29; *p* < 0.01; *d* = 1.85). - Positive aspects of using AI-based system included: easy access, empathy, friendly interesting, educational, exploring depression, interactive, and choice list. - Negative comments regarding chatbot use were impersonal, unnatural, rigid patterns, misunderstanding, repetitive contents, too general, irrelevant contents, or too simple.

**Table 3:** Quality assessment of Before-After Studies.

	[77]	[81]	[82]	[83]
1. Was the study question or objective clearly stated?	Yes	Yes	Yes	Yes
2. Were eligibility/selection criteria for the study population prespecified and clearly described?	Yes	Yes	Yes	Yes
3. Were the participants in the study representative of those who would be eligible for the test/service/intervention in the general or clinical population of interest?	Yes	Yes	Yes	Yes
4. Were all eligible participants that met the prespecified entry criteria enrolled?	No	Yes	Yes	Yes
5. Was the sample size sufficiently large to provide confidence in the findings?	NR	Yes	Yes	NR
6. Was the test/service/intervention clearly described and delivered consistently across the study population?	Yes	Yes	No	Yes
7. Were the outcome measures prespecified, clearly defined, valid, reliable, and assessed consistently across all study participants?	Yes	Yes	Yes	Yes
8. Were the people assessing the outcomes blinded to the participants’ exposures/interventions?	NA	Yes	NA	NA
9. Was the loss to follow-up after baseline 20% or less? Were those lost to follow-up accounted for in the analysis?	Yes	Yes	No/Yes	No/No
10. Did the statistical methods examine changes in outcome measures from before to after the intervention? Were statistical tests done that provided p values for the pre-to-post changes?	Yes	Yes	Yes	Yes
11. Were outcome measures of interest taken multiple times before the intervention and multiple times after the intervention (i.e., did they use an interrupted time-series design)?	Yes	No	Yes	Yes
12. If the intervention was conducted at a group level (e.g., a whole hospital, a community, etc.) did the statistical analysis consider the use of individual-level data to determine effects at the group level?	NA	NA	NA	NA
**Total score (maximum 12 points)**	**8**	**10**	**9**	**8**

Note: CD, cannot determine; NA, not applicable; NR, not reported.

**Table 4:** Quality assessment of Case Series Studies.

	[85]
1. Was the study question or objective clearly stated?	Yes
2. Was the study population clearly and fully described, including a case definition?	Yes
3. Were the cases consecutive?	Yes
4. Were the subjects comparable?	Yes
5. Was the intervention clearly described?	Yes
6. Were the outcome measures clearly defined, valid, reliable, and implemented consistently across all study participants?	Yes
7. Was the length of follow-up adequate?	NA
8. Were the statistical methods well-described?	Yes
9. Were the results well-described?	Yes
**Total score (maximum 9 points)**	**8**

Note: CD, cannot determine; NA, not applicable; NR, not reported.

**Table 5:** Quality assessment of Case-Control Studies.

	[84]
1. Was the research question or objective in this paper clearly stated and appropriate?	Yes
2. Was the study population clearly specified and defined?	Yes
3. Did the authors include a sample size justification?	No
4. Were controls selected or recruited from the same or similar population that gave rise to the cases (including the same timeframe)?	Yes
5. Were the definitions, inclusion and exclusion criteria, algorithms or processes used to identify or select cases and controls valid, reliable, and implemented consistently across all study participants?	CD
6. Were the cases clearly defined and differentiated from controls?	Yes
7. If less than 100 percent of eligible cases and/or controls were selected for the study, were the cases and/or controls randomly selected from those eligible?	NR
8. Was there use of concurrent controls?	No
9. Were the investigators able to confirm that the exposure/risk occurred prior to the development of the condition or event that defined a participant as a case?	Yes
10. Were the measures of exposure/risk clearly defined, valid, reliable, and implemented consistently (including the same time period) across all study participants?	Yes
11. Were the assessors of exposure/risk blinded to the case or control status of participants?	No
12. Were key potential confounding variables measured and adjusted statistically in the analyses? If matching was used, did the investigators account for matching during study analysis?	Yes
**Total score (maximum 12 points)**	**7**

Note: CD, cannot determine; NA, not applicable; NR, not reported.

**Table 6:** Quality assessment of Controlled Intervention Studies.

	[79]	[80]	[78]	[86]
1. Was the study described as randomized, a randomized trial, a randomized clinical trial, or an RCT?	No	Yes	Yes	Yes
2. Was the method of randomization adequate (i.e., use of randomly generated assignment)?	NR	Yes	Yes	Yes
3. Was the treatment allocation concealed (so that assignments could not be predicted)?	NR	Yes	Yes	Yes
4. Were study participants and providers blinded to treatment group assignment?	NR	NR	Yes	No
5. Were the people assessing the outcomes blinded to the participants’ group assignments?	Yes	Yes	Yes	No
6. Were the groups similar at baseline on important characteristics that could affect outcomes (e.g., demographics, risk factors, or co-morbid conditions)?	NR	No	Yes	Yes
7. Was the overall drop-out rate from the study at endpoint 20% or lower of the number allocated to treatment?	NR	No	Yes	No
8. Was the differential drop-out rate (between treatment groups) at endpoint 15 percentage points or lower?	NR	Yes	Yes	Yes
9. Was there high adherence to the intervention protocols for each treatment group?	NR	Yes	Yes	Yes
10. Were other interventions avoided or similar in the groups (e.g., similar background treatments)?	NR	NR	Yes	Yes
11. Were outcomes assessed using valid and reliable measures, implemented consistently across all study participants?	Yes	No	Yes	Yes
12. Did the authors report that the sample size was sufficiently large to be able to detect a difference in the main outcome between groups with at least 80% power?	No	Yes	Yes	Yes
13. Were outcomes reported or subgroups analyzed prespecified (i.e., identified before analyses were conducted)?	No	Yes	Yes	Yes
14. Were all randomized participants analyzed in the group to which they were originally assigned, i.e., did they use an intention-to-treat analysis?	NR	No	Yes	Yes
**Total score (maximum 14 points)**	**2**	**8**	**14**	**9**

Note: CD, cannot determine; NA, not applicable; NR, not reported.

## Supplementary Materials

The following supporting information can be downloaded at: https://www.mdpi.com/article/10.3390/ijerph19137737/s1.

## References

[1] Bullis J.R., Boettcher H., Sauer-Zavala S., Barlow D.H. (2019). What Is an Emotional Disorder? A Transdiagnostic Mechanistic Definition with Implications for Assessment, Treatment, and Prevention. Clin. Psychol. Sci. Pract..

[2] Baxter A.J., Scott K.M., Ferrari A., Norman R.E., Vos T., Whiteford H. (2014). Challenging the myth of an “epidemic” of common mental disorders: Trends in the global prevalence of anxiety and depression between 1990 and 2010. Depress. Anxiety.

[3] Lim G.Y., Tam W.W., Lu Y., Ho C.S., Zhang M.W., Ho R.C. (2018). Prevalence of Depression in the Community from 30 Countries between 1994 and 2014. Sci. Rep..

[4] Boyd A., Van de Velde S., Vilagut G., de Graaf R., O’Neill S., Florescu S., Alonso J., Kovess-Masfety V. (2015). Gender Differences in Mental Disorders and Suicidality in Europe: Results from a Large Cross-Sectional Population-Based Study. J. Affect. Disord..

[5] Wittchen H.U., Jacobi F., Rehm J., Gustavsson A., Svensson M., Jönsson B., Olesen J., Allgulander C., Alonso J., Faravelli C. (2011). The Size and Burden of Mental Disorders and Other Disorders of the Brain in Europe 2010. Eur. Neuropsychopharmacol..

[6] González-Blanch C., Umaran-Alfageme O., Cordero-Andrés P., Muñoz-Navarro R., Ruiz-Rodríguez P., Medrano L.A., Ruiz-Torres M., Dongil Collado E., Cano-Vindel A. (2018). Tratamiento Psicológico de Los Trastornos Emocionales En Atención Primaria: El Manual de Tratamiento Transdiagnóstico Del Estudio PsicAP. Ansiedad Estrés.

[7] Goldberg D.P., Reed G.M., Robles R., Minhas F., Razzaque B., Fortes S., de Jesus Mari J., Lam T.P., Garcia J.Á., Gask L. (2017). Screening for Anxiety, Depression, and Anxious Depression in Primary Care: A Field Study for ICD-11 PHC. J. Affect. Disord..

[8] Vindel A.C., Salguero J.M., Wood C.M., Dongil E., Latorre J.M., Antonio C., Vindel C. (2012). La Depresión En Atención Primaria: Prevalencia, Diagnóstico y Tratamiento. Pap. Psicólogo.

[9] King M., Nazareth I., Levy G., Walker C., Morris R., Weich S., Bellón-Saameño J.Á., Moreno B., Švab I., Rotar D. (2008). Prevalence of Common Mental Disorders in General Practice Attendees across Europe. Br. J. Psychiatry.

[10] Serrano-Blanco A., Palao D.J., Luciano J.V., Pinto-Meza A., Luján L., Fernández A., Roura P., Bertsch J., Mercader M., Haro J.M. (2010). Prevalence of Mental Disorders in Primary Care: Results from the Diagnosis and Treatment of Mental Disorders in Primary Care Study (DASMAP). Soc. Psychiatry Psychiatr. Epidemiol..

[11] World Health Assembly (2012). Global Burden of Mental Disorders and the Need for a Comprehensive, Coordinated Response from Health and Social Sectors at the Country Level.

[12] Knapp M., Wong G. (2020). Economics and Mental Health: The Current Scenario. World Psychiatry.

[13] Harvey A.G., Gumport N.B. (2015). Evidence-Based Psychological Treatments for Mental Disorders: Modifiable Barriers to Access and Possible Solutions. Behav. Res. Ther..

[14] Wang P.S., Aguilar-Gaxiola S., Alonso J., Angermeyer M.C., Borges G., Bromet E.J., Bruffaerts R., de Girolamo G., de Graaf R., Gureje O. (2007). Use of Mental Health Services for Anxiety, Mood, and Substance Disorders in 17 Countries in the WHO World Mental Health Surveys. Lancet.

[15] Samele C., Frew S., Urquía N. (2013). Mental Health Systems in the European Union Member States, Status of Mental Health in Populations and Benefits to Be Expected from Investments into Mental Health.

[16] Purebl G., Petrea I., Shields L., Tóth M.D., Székely A., Kurimay T., McDaid D., Arensman E., Granic I., Abello K.M. (2015). Joint Action on Mental Health and Well-Being: Situation Analysis and Recommendations for Action.

[17] Barbato A., Vallarino M., Rapisarda F., Lora A., Caldas de Almeida J.M. (2016). Access to Mental Health Care in Europe. https://www.researchgate.net/publication/319329050_Access_to_Mental_Health_Care_in_Europe.

[18] Pikouli Κ., Konstantakopoulos G., Kalampaka Spilioti P., Fytrolaki E., Ploumpidis D., Economou M. (2019). The Impact of the Recent Financial Crisis on the Users’ Profile of a Community Mental Health Unit. Psychiatriki.

[19] Wampold B.E., Budge S.L., Laska K.M., Del Re A.C., Baardseth T.P., Flűckiger C., Minami T., Kivlighan D.M., Gunn W. (2011). Evidence-Based Treatments for Depression and Anxiety versus Treatment-as-Usual: A Meta-Analysis of Direct Comparisons. Clin. Psychol. Rev..

[20] Bandelow B., Michaelis S., Wedekind D. (2017). Treatment of Anxiety Disorders. Dialogues Clin. Neurosci..

[21] American Psychological Association (2019). Clinical Practice Guideline for the Treatment of Depression across Three Age Cohorts.

[22] Katzman M.A., Bleau P., Blier P., Chokka P., Kjernisted K., Van Ameringen M., Antony M.M., Bouchard S., Brunet A., Flament M. (2014). Canadian Clinical Practice Guidelines for the Management of Anxiety, Posttraumatic Stress and Obsessive-Compulsive Disorders. BMC Psychiatry.

[23] Gautam S., Jain A., Gautam M., Vahia V., Grover S. (2017). Clinical Practice Guidelines for the Management of Depression. Indian J. Psychiatry.

[24] Sher L. (2020). The Impact of the COVID-19 Pandemic on Suicide Rates. QJM.

[25] Moreno C., Wykes T., Galderisi S., Nordentoft M., Crossley N., Jones N., Cannon M., Correll C.U., Byrne L., Carr S. (2020). How Mental Health Care Should Change as a Consequence of the COVID-19 Pandemic. Lancet Psychiatry.

[26] Salari N., Hosseinian-Far A., Jalali R., Vaisi-Raygani A., Rasoulpoor S., Mohammadi M., Rasoulpoor S., Khaledi-Paveh B. (2020). Prevalence of Stress, Anxiety, Depression among the General Population during the COVID-19 Pandemic: A Systematic Review and Meta-Analysis. Global. Health.

[27] Ettman C.K., Abdalla S.M., Cohen G.H., Sampson L., Vivier P.M., Galea S. (2020). Prevalence of Depression Symptoms in US Adults Before and During the COVID-19 Pandemic. JAMA Netw. Open.

[28] Fujiwara D., Dolan P., Lawton R., Behzadnejad F., Lagarde A., Maxwell C., Peytrignet S. (2020). The Wellbeing Costs of COVID-19 in the UK; Report by Simetrica-Jacobs and the London School of Economics and Political Science. https://www.ceci.org.uk/wp-content/uploads/2020/05/jacobs-wellbeing-costs-of-covid-19-uk.pdf.

[29] McGinty E.E., Presskreischer R., Han H., Barry C.L. (2020). Psychological Distress and Loneliness Reported by US Adults in 2018 and April 2020. JAMA.

[30] Lake J., Turner M.S. (2017). Urgent Need for Improved Mental Health Care and a More Collaborative Model of Care. Perm. J..

[31] Kazdin A.E. (2015). Treatment as Usual and Routine Care in Research and Clinical Practice. Clin. Psychol. Rev..

[32] Clark D.M. (2011). Implementing NICE Guidelines for the Psychological Treatment of Depression and Anxiety Disorders: The IAPT Experience. Int. Rev. Psychiatry.

[33] Clark D.M. (2018). Realizing the Mass Public Benefit of Evidence-Based Psychological Therapies: The IAPT Program. Annu. Rev. Clin. Psychol..

[34] Mohr D.C., Weingardt K.R., Reddy M., Schueller S.M. (2017). Three Problems with Current Digital Mental Health Research and Three Things We Can Do About Them. Psychiatr. Serv..

[35] Miralles I., Granell C., Díaz-Sanahuja L., van Woensel W., Bretón-López J., Mira A., Castilla D., Casteleyn S. (2020). Smartphone Apps for the Treatment of Mental Disorders: Systematic Review. JMIR mHealth uHealth.

[36] Andersson G. (2016). Internet-Delivered Psychological Treatments. Annu. Rev. Clin. Psychol..

[37] Karyotaki E., Ebert D.D., Donkin L., Riper H., Twisk J., Burger S., Rozental A., Lange A., Williams A.D., Zarski A.C. (2018). Do Guided Internet-Based Interventions Result in Clinically Relevant Changes for Patients with Depression? An Individual Participant Data Meta-Analysis. Clin. Psychol. Rev..

[38] Andrews G., Basu A., Cuijpers P., Craske M.G., McEvoy P., English C.L., Newby J.M. (2018). Computer Therapy for the Anxiety and Depression Disorders Is Effective, Acceptable and Practical Health Care: An Updated Meta-Analysis. J. Anxiety Disord..

[39] Carlbring P., Andersson G., Cuijpers P., Riper H., Hedman-Lagerlöf E. (2018). Internet-Based vs. Face-to-Face Cognitive Behavior Therapy for Psychiatric and Somatic Disorders: An Updated Systematic Review and Meta-Analysis. Cogn. Behav. Ther..

[40] Richards D., Enrique A., Eilert N., Franklin M., Palacios J., Duffy D., Earley C., Chapman J., Jell G., Sollesse S. (2020). A Pragmatic Randomized Waitlist-Controlled Effectiveness and Cost-Effectiveness Trial of Digital Interventions for Depression and Anxiety. NPJ Digit. Med..

[41] Massoudi B., Holvast F., Bockting C.L.H., Burger H., Blanker M.H. (2019). The Effectiveness and Cost-Effectiveness of e-Health Interventions for Depression and Anxiety in Primary Care: A Systematic Review and Meta-Analysis. J. Affect. Disord..

[42] Mehta S., Peynenburg V.A., Hadjistavropoulos H.D. (2019). Internet-Delivered Cognitive Behaviour Therapy for Chronic Health Conditions: A Systematic Review and Meta-Analysis. J. Behav. Med..

[43] Andrews G., Williams A.D. (2015). Up-Scaling Clinician Assisted Internet Cognitive Behavioural Therapy (ICBT) for Depression: A Model for Dissemination into Primary Care. Clin. Psychol. Rev..

[44] Andersson G., Carlbring P., Titov N., Lindefors N. (2019). Internet Interventions for Adults with Anxiety and Mood Disorders: A Narrative Umbrella Review of Recent Meta-Analyses. Can. J. Psychiatry.

[45] Lambert M.J., Norcross J.C., VandenBos G.R., Freedheim D.K. (2011). Psychotherapy Research and Its Achievements. History of Psychotherapy: Continuity and Change.

[46] Barth J., Munder T., Gerger H., Nüesch E., Trelle S., Znoj H., Jüni P., Cuijpers P. (2013). Comparative Efficacy of Seven Psychotherapeutic Interventions for Patients with Depression: A Network Meta-Analysis. PLoS Med..

[47] Zhou X., Hetrick S.E., Cuijpers P., Qin B., Barth J., Whittington C.J., Cohen D., Del Giovane C., Liu Y., Michael K.D. (2015). Comparative Efficacy and Acceptability of Psychotherapies for Depression in Children and Adolescents: A Systematic Review and Network Meta-Analysis. World Psychiatry.

[48] Kravitz R.L., Duan N., Niedzinski E.J., Hay M.C., Subramanian S.K., Weisner T.S. (2008). What Ever Happened to N-of-1 Trials? Insiders’ Perspectives and a Look to the Future. Milbank Q..

[49] Ebert D.D., Gollwitzer M., Riper H., Cuijpers P., Baumeister H., Berking M. (2013). For Whom Does It Work? Moderators of Outcome on the Effect of a Transdiagnostic Internet-Based Maintenance Treatment After Inpatient Psychotherapy: Randomized Controlled Trial. J. Med. Internet Res..

[50] Cuijpers P., Reijnders M., Huibers M.J.H. (2019). The Role of Common Factors in Psychotherapy Outcomes. Annu. Rev. Clin. Psychol..

[51] Evans J.G. (1995). Evidence-Based and Evidence-Biased Medicine. Age Ageing.

[52] Fonagy P. (2015). The Effectiveness of Psychodynamic Psychotherapies: An Update. World Psychiatry.

[53] Beutler L.E., Kimpara S., Edwards C.J., Miller K.D. (2018). Fitting Psychotherapy to Patient Coping Style: A Meta-Analysis. J. Clin. Psychol..

[54] Paul G.L. (1967). Strategy of Outcome Research in Psychotherapy. J. Consult. Psychol..

[55] Chorpita B.F., Rotheram-Borus M.J., Daleiden E.L., Bernstein A., Cromley T., Swendeman D., Regan J. (2011). The Old Solutions Are the New Problem. Perspect. Psychol. Sci..

[56] Păsărelu C.R., Andersson G., Bergman Nordgren L., Dobrean A. (2017). Internet-Delivered Transdiagnostic and Tailored Cognitive Behavioral Therapy for Anxiety and Depression: A Systematic Review and Meta-Analysis of Randomized Controlled Trials. Cogn. Behav. Ther..

[57] Gallo K.P., Barlow D.H. (2012). Factors Involved in Clinician Adoption and Nonadoption of Evidence-based Interventions in Mental Health. Clin. Psychol. Sci. Pract..

[58] Cook S.C., Schwartz A.C., Kaslow N.J. (2017). Evidence-Based Psychotherapy: Advantages and Challenges. Neurotherapeutics.

[59] Myin-Germeys I., Klippel A., Steinhart H., Reininghaus U. (2016). Ecological Momentary Interventions in Psychiatry. Curr. Opin. Psychiatry.

[60] Heron K.E., Smyth J.M. (2010). Ecological Momentary Interventions: Incorporating Mobile Technology into Psychosocial and Health Behaviour Treatments. Br. J. Health Psychol..

[61] Gee B.L., Griffiths K.M., Gulliver A. (2016). Effectiveness of Mobile Technologies Delivering Ecological Momentary Interventions for Stress and Anxiety: A Systematic Review. J. Am. Med. Inform. Assoc..

[62] Olff M. (2015). Mobile Mental Health: A Challenging Research Agenda. Eur. J. Psychotraumatol..

[63] Price M., Yuen E.K., Goetter E.M., Herbert J.D., Forman E.M., Acierno R., Ruggiero K.J. (2014). MHealth: A Mechanism to Deliver More Accessible, More Effective Mental Health Care. Clin. Psychol. Psychother..

[64] Kazdin A.E. (2014). Moderators, Mediators and Mechanisms of Change in Psychotherapy. Quantitative and Qualitative Methods in Psychotherapy Research.

[65] Donker T., Batterham P.J., Warmerdam L., Bennett K., Bennett A., Cuijpers P., Griffiths K.M., Christensen H. (2013). Predictors and Moderators of Response to Internet-Delivered Interpersonal Psychotherapy and Cognitive Behavior Therapy for Depression. J. Affect. Disord..

[66] Kazdin A.E. (2007). Mediators and Mechanisms of Change in Psychotherapy Research. Annu. Rev. Clin. Psychol..

[67] Luxton D.D. (2014). Artificial Intelligence in Psychological Practice: Current and Future Applications and Implications. Prof. Psychol. Res. Pract..

[68] Bach Xuan T., Giang Thu V., Giang Hai H., Quan-Hoang V., Manh-Tung H., Thu-Trang V., Viet-Phuong L., Manh-Toan H., Nghiem K.-C.P., Huong Lan Thi N. (2019). Global Evolution of Research in Artificial Intelligence in Health and Medicine: A Bibliometric Study. J. Clin. Med..

[69] De Mello F.L., de Souza S.A. (2019). Psychotherapy and Artificial Intelligence: A Proposal for Alignment. Front. Psychol..

[70] Horn R.L., Weisz J.R. (2020). Can Artificial Intelligence Improve Psychotherapy Research and Practice?. Adm. Policy Ment. Health Ment. Health Serv. Res..

[71] Pintelas E.G., Kotsilieris T., Livieris I.E., Pintelas P. (2018). A Review of Machine Learning Prediction Methods for Anxiety Disorders. Proceedings of the 8th International Conference on Software Development and Technologies for Enhancing Accessibility and Fighting Info-Exclusion-DSAI 2018.

[72] Aafjes-van Doorn K., Kamsteeg C., Bate J., Aafjes M. (2021). A Scoping Review of Machine Learning in Psychotherapy Research. Psychother. Res..

[73] Suso-Ribera C., Castilla D., Martínez-Borba V., Jaén I., Botella C., Baños R.M., García-Palacios A. (2022). Technological Interventions for Pain Management. Ref. Modul. Neurosci. Biobehav. Psychol..

[74] Legler S., Celano C.M., Amador A., Novis A., Ebrahim S., Huffman J.C. (2018). Development and Theoretical Approach to an Adaptive Text Message Program to Promote Well-Being and Health Behaviors in Primary Care Patients. Prim. Care Companion CNS Disord..

[75] Kelly J., Gooding P., Pratt D., Ainsworth J., Welford M., Tarrier N. (2012). Intelligent Real-Time Therapy: Harnessing the Power of Machine Learning to Optimise the Delivery of Momentary Cognitivebehavioural Interventions. J. Ment. Health.

[76] Moher D., Liberati A., Tetzlaff J., Altman D.G., Altman D.G., Antes G., Atkins D., Barbour V., Barrowman N., Berlin J.A. (2009). Preferred Reporting Items for Systematic Reviews and Meta-Analyses: The PRISMA Statement (Chinese Edition). J. Chin. Integr. Med..

[77] Burns M.N., Begale M., Duffecy J., Gergle D., Karr C.J., Giangrande E., Mohr D.C. (2011). Harnessing Context Sensing to Develop a Mobile Intervention for Depression. J. Med. Internet Res..

[78] Fulmer R., Joerin A., Gentile B., Lakerink L., Rauws M. (2018). Using Psychological Artificial Intelligence (Tess) to Relieve Symptoms of Depression and Anxiety: Randomized Controlled Trial. JMIR Ment. Health.

[79] Paredes P., Berkeley U.C., Gilad-bachrach R., Czerwinski M., Roseway A., Hernandez J. PopTherapy: Coping with Stress through Pop-Culture. Proceedings of the 8th International Conference on Pervasive Computing Technologies for Healthcare.

[80] Bird T., Mansell W., Wright J., Gaffney H., Tai S. (2018). Manage Your Life Online: A Web-Based Randomized Controlled Trial Evaluating the Effectiveness of a Problem-Solving Intervention in a Student Sample. Behav. Cogn. Psychother..

[81] Inkster B., Sarda S., Subramanian V. (2018). An Empathy-Driven, Conversational Artificial Intelligence Agent (Wysa) for Digital Mental Well-Being: Real-World Data Evaluation Mixed-Methods Study. JMIR mHealth uHealth.

[82] Lutz W., Rubel J.A., Schwartz B., Schilling V., Deisenhofer A.K. (2019). Towards Integrating Personalized Feedback Research into Clinical Practice: Development of the Trier Treatment Navigator (TTN). Behav. Res. Ther..

[83] Wahle F., Kowatsch T., Fleisch E., Rufer M., Weidt S. (2016). Mobile Sensing and Support for People with Depression: A Pilot Trial in the Wild. JMIR mHealth uHealth.

[84] Cho C.H., Lee T., Lee J.B., Seo J.Y., Jee H.J., Son S., An H., Kim L., Lee H.J. (2020). Effectiveness of a Smartphone App with a Wearable Activity Tracker in Preventing the Recurrence of Mood Disorders: Prospective Case-Control Study. JMIR Ment. Health.

[85] Green E.P., Lai Y., Pearson N., Rajasekharan S., Rauws M., Joerin A., Kwobah E., Musyimi C., Jones R.M., Bhat C. (2020). Expanding Access to Perinatal Depression Treatment in Kenya through Automated Psychological Support: Development and Usability Study. JMIR Form. Res..

[86] Liu H., Peng H., Song X., Xu C., Zhang M. (2022). Using AI Chatbots to Provide Self-Help Depression Interventions for University Students: A Randomized Trial of Effectiveness. Internet Interv..

[87] Taubitz F.-S., Büdenbender B., Alpers G.W. (2022). What the Future Holds: Machine Learning to Predict Success in Psychotherapy. Behav. Res. Ther..

[88] Russell S., Norvig P. (2009). Artificial Intelligence: A Modern Approach.

[89] Miner A.S., Shah N., Bullock K.D., Arnow B.A., Bailenson J., Hancock J. (2019). Key Considerations for Incorporating Conversational AI in Psychotherapy. Front. Psychiatry.

[90] Sedlakova J., Trachsel M. (2022). Conversational Artificial Intelligence in Psychotherapy: A New Therapeutic Tool or Agent?. Am. J. Bioeth..

