# Supplementary material for: Using Artificial Intelligence to Enhance Ongoing Psychological Interventions for Emotional Problems in Real- or Close to Real-Time: A Systematic Review
Source: Int J Environ Res Public Health. 2022 Jun 24;19(13):7737. doi: 10.3390/ijerph19137737 (PMC9266240; doi:10.3390/ijerph19137737)
Supplement: Supplementary file 1 [file ijerph-19-07737-s001.zip › ijerph-1743160-supplementary.pdf]

Supplementary File S1. Search strategy used for each electronic database

Web of Science

AB=("Mood Disorder" OR "Mood Disorders" OR "Emotional Disorder" OR "Emotional Disorders" OR anxiety OR Depressive OR Depression OR "Bipolar Disorder" OR "Bipolar Disorders" OR ptsd OR “posttraumatic stress” OR “post-traumatic stress” OR ocd OR “obsessive-compulsive” OR “obsessive compulsive” OR phobia OR phobias OR panic OR agoraphobia OR dysthymia OR “dysthymic disorder”)
AND AB=("artificial intelligence" OR "machine learning")
AND AB=(treatment OR intervention OR therapy OR psychotherapy)

TI=("Mood Disorder" OR "Mood Disorders" OR "Emotional Disorder" OR "Emotional Disorders" OR anxiety OR Depressive OR Depression OR "Bipolar Disorder" OR "Bipolar Disorders" OR ptsd OR “posttraumatic stress” OR “post-traumatic stress” OR ocd OR “obsessive-compulsive” OR “obsessive compulsive” OR phobia OR phobias OR panic OR agoraphobia OR dysthymia OR “dysthymic disorder”)
AND TI=("artificial intelligence" OR "machine learning")
AND TI=(treatment OR intervention OR therapy OR psychotherapy)

Pubmed

((mood disorder[Title/Abstract]) OR (mood disorders[Title/Abstract]) OR (emotional disorder[Title/Abstract]) OR (emotional disorders[Title/Abstract]) OR (anxiety[Title/Abstract]) OR (depression[Title/Abstract]) OR (depressive[Title/Abstract]) OR (bipolar disorder[Title/Abstract]) OR (bipolar disorders[Title/Abstract]) OR (PTSD[Title/Abstract]) OR (posttraumatic stress[Title/Abstract]) OR (post-traumatic stress[Title/Abstract]) OR (OCD[Title/Abstract]) OR (obsessive-compulsive[Title/Abstract]) OR (obsessive compulsive[Title/Abstract]) OR (phobia[Title/Abstract]) OR (phobias[Title/Abstract]) OR (agoraphobia[Title/Abstract]) OR (dysthymia[Title/Abstract]) OR (dysthymic disorder[Title/Abstract])) AND ((machine learning[Title/Abstract]) OR (artificial intelligence[Title/Abstract])) AND ((psychotherapy[Title/Abstract]) OR (treatment[Title/Abstract]) OR (intervention[Title/Abstract]) OR (therapy[Title/Abstract]) OR (psychological[Title/Abstract]))

Scopus

((TITLE-ABS-KEY("Mood Disorder") OR TITLE-ABS-KEY("Mood Disorders") OR TITLE-ABS-KEY("Emotional Disorder") OR TITLE-ABS-KEY("Emotional Disorders") OR TITLE-ABS-KEY(anxiety) OR TITLE-ABS-KEY(depressive) OR TITLE-ABS-KEY(depression) OR TITLE-ABS-KEY(“bipolar disorder”) OR TITLE-ABS-KEY(“bipolar disorders”) OR TITLE-ABS-KEY(ptsd) OR TITLE-ABS-KEY(“post-traumatic stress”) OR TITLE-ABS-KEY(“posttraumatic stress") OR TITLE-ABS-KEY(ocd) OR TITLE-ABS-KEY(“obsessive-compulsive”) OR TITLE-ABS-KEY(“obsessive compulsive”) OR TITLE-ABS-KEY(phobia) OR TITLE-ABS-KEY(phobias) OR TITLE-ABS-KEY(panic) OR TITLE-ABS-KEY(agoraphobia) OR TITLE-ABS-KEY(dysthymia) OR TITLE-ABS-KEY(“dysthymic disorder”))

AND

(TITLE-ABS-KEY(treatment) OR TITLE-ABS-KEY(intervention) OR TITLE-ABS-KEY(therapy) OR TITLE-ABS-KEY(psychotherapy))

AND

(TITLE-ABS-KEY("artificial intelligence") OR TITLE-ABS-KEY("machine learning")))

Cochrane:

(("mood disorder"):ti,ab,kw OR ("mood disorders"):ti,ab,kw OR ("emotional disorder"):ti,ab,kw OR ("emotional disorders"):ti,ab,kw OR ("anxiety"):ti,ab,kw OR ("depressive"):ti,ab,kw OR ("depression"):ti,ab,kw OR ("bipolar disorder"):ti,ab,kw OR ("bipolar disorders"):ti,ab,kw OR ("ptsd"):ti,ab,kw OR (“post-traumatic stress”):ti,ab,kw OR (“posttraumatic stress”):ti,ab,kw OR ("ocd"):ti,ab,kw OR ("obsessive-compulsive"):ti,ab,kw OR ("obsessive compulsive"):ti,ab,kw OR ("phobia"):ti,ab,kw OR ("phobias"):ti,ab,kw OR ("panic"):ti,ab,kw OR ("agoraphobia"):ti,ab,kw OR ("dysthymia"):ti,ab,kw OR ("dysthymic disorder"):ti,ab,kw)

AND

(("machine learning"):ti,ab,kw OR ("artificial intelligence"):ti,ab,kw)

AND

(("treatment"):ti,ab,kw OR ("intervention"):ti,ab,kw OR ("therapy"):ti,ab,kw OR ("psychotherapy"):ti,ab,kw)
